# Comparison of an automated smartphone-based smoking cessation intervention versus standard quitline-delivered treatment among underserved smokers: protocol for a randomized controlled trial

**DOI:** 10.1186/s12889-022-12840-7

**Published:** 2022-03-22

**Authors:** Jennifer I. Vidrine, Ya-Chen Tina Shih, Michael S. Businelle, Steven K. Sutton, Diana Stewart Hoover, Cherell Cottrell-Daniels, Bethany Shorey Fennell, Kristina E. Bowles, Damon J. Vidrine

**Affiliations:** 1 Department of Health Outcomes and Behavior, Moffitt Cancer Center, 4115 E. Fowler Avenue, Tampa, FL 33617 USA; 2 Department of Oncologic Sciences, University of South Florida, Morsani College of Medicine, Tampa, FL USA; 3 Department of Psychology, University of South Florida, Tampa, FL USA; 4 Department of Health Services Research, University of Texas MD Anderson Cancer Center, Houston, TX USA; 5 Stephenson Cancer Center, TSET Health Promotion Research Center, University of Oklahoma Health Sciences Center, Oklahoma City, OK USA; 6 Department of Biostatistics and Bioinformatics, Moffitt Cancer Center, Tampa, FL USA; 7 Hoover Editing, Asheville, NC USA

**Keywords:** Smoking cessation, Low-socioeconomic status, Health disparities, mHealth

## Abstract

**Background:**

Smoking is the leading cause of preventable morbidity and mortality in the United States. Individuals with low socioeconomic status have disproportionately high smoking rates and greater difficulty quitting smoking. Efficiently connecting underserved smokers to effective tobacco cessation programs is crucial for disease prevention and the elimination of health disparities. Smartphone-based interventions have the potential to enhance the reach and efficacy of smoking cessation treatments targeting underserved smokers, but there is little efficacy data for these interventions. In this study, we will partner with a large, local hunger-relief organization to evaluate the efficacy and economic impact of a theoretically-based, fully-automated, and interactive smartphone-based smoking cessation intervention.

**Methods:**

This study will consist of a 2-group randomized controlled trial. Participants (*N* = 500) will be recruited from a network of food distribution centers in West Central Florida and randomized to receive either Standard Treatment (ST, *n* = 250) or Automated Treatment (AT, *n* = 250). ST participants will be connected to the Florida Quitline for telephone-based treatment and will receive a 10-week supply of nicotine replacement therapy (NRT; transdermal patches and lozenges). AT participants will receive 10 weeks of NRT and a fully-automated smartphone-based intervention consisting of interactive messaging, images, and audiovisual clips. The AT intervention period will span 26 weeks, with 12 weeks of proactive content and 26 weeks of on-demand access. ST and AT participants will complete weekly 4-item assessments for 26 weeks and 3-, 6-, and 12-month follow-up assessments. Our primary aim is to evaluate the efficacy of AT in facilitating smoking abstinence. As secondary aims, we will explore potential mediators and conduct economic evaluations to assess the cost and/or cost-effectiveness of ST vs. AT.

**Discussion:**

The overall goal of this project is to determine if AT is better at facilitating long-term smoking abstinence than ST, the more resource-intensive approach. If efficacy is established, the AT approach will be relatively easy to disseminate and for community-based organizations to scale and implement, thus helping to reduce tobacco-related health disparities.

**Trial registration:**

Clinical Trials Registry NCT05004662. Registered August 13, 2021.

## Background

In the United States (US), smoking is the leading cause of preventable morbidity and mortality [1]. Although overall smoking rates have declined in recent years, smoking is most concentrated among adults with the lowest levels of education, income, and occupational status—or those with low socioeconomic status (SES; [2]). Low-SES smokers are just as likely as those with higher SES to make a quit attempt [1], but they have greater difficulty quitting and remaining abstinent [3]. This is at least partially because low-SES smokers have less access to evidence-based and effective smoking cessation resources and are less likely to use such resources [4]. Thus, cigarette smoking accounts for a significant proportion of socioeconomic disparities in the incidence and mortality of disease [1, 5, 6]. Efforts are needed to connect low-SES smokers with effective, accessible, and engaging smoking cessation treatments to prevent disease and eliminate tobacco-related health disparities.

### Quitline-delivered smoking cessation treatment

Our team has developed partnerships with several safety-net hospitals and HIV clinics to identify and connect underserved smokers with evidence-based smoking cessation treatment delivered via state quitlines [7–10]. We developed Ask Advise Connect (AAC) to help link smokers with treatment via an automated connection system within the electronic health record. AAC has demonstrated tremendous potential as an efficient means for helping smokers obtain evidence-based treatment [7–10]; however, interventions such as AAC that depend on connecting smokers in healthcare settings with quitlines may not be adequate for low SES smokers. National data indicate that smokers and individuals with low SES do not regularly visit healthcare providers [3, 11, 12], and when they do, they are not regularly asked about their smoking status, advised to quit smoking, or offered help with quitting [13]. Moreover, in recent years, many state quitlines have experienced budget cuts, forcing them to limit or suspend services [14–16]. Phone-based counseling also has limited appeal, as quitlines reach only 1–2% of smokers [17]. In order to adequately reach underserved smokers, efforts are needed to develop and improve access to evidence-based treatments that can be delivered in the community.

### Mobile technology and smoking cessation treatment

In the US, smartphone ownership is widespread. In April 2021, the Pew Research Center reported that 85% of US adults own a smartphone [18]. Smartphone ownership is high among individuals between the ages of 18–64 years (83%) and racial/ethnic minorities (83%). It is also high among individuals with less than a high school education (75%) and an annual household income of less than $30,000 (76%) as well as those living in rural communities (80%). Additionally, the Pew Research Center reported that many US adults depend on their smartphones for all internet access. This proportion is high among racial/ethnic minority groups (vs. whites), individuals with lower education and income, and those living in rural communities. These trends suggest that smartphone-delivered interventions might be an excellent method for reaching smokers from underserved populations (e.g., low SES, rural, racial/ethnic minority).

Prior studies have used cell phones to administer text message-based, or short message service (SMS), smoking cessation interventions. Results suggest that these interventions are efficacious [19–21] and cost-effective [22, 23]. In fact, text message-based smoking cessation interventions are one of the most affordable interventions for global tobacco control [24], and these interventions have been endorsed by various organizations, including the World Health Organization [25, 26]. Smartphones have greater capability than cell phones and can be used to access the internet, run applications (apps), view and send graphic messages, and stream audiovisual content. Smartphone-delivered interventions, using mobile websites and apps, have been developed; yet many of these treatments do not adhere to the evidence-based practices established by the Treating Tobacco Use and Dependence Clinical Practice Guideline, and there is surprisingly little efficacy data for these interventions [27, 28]. Such evidence is particularly lacking among low-SES smokers [29]. Given the ubiquity of smartphones, interventions utilizing smartphones have the potential to enhance the reach and efficacy of smoking cessation interventions targeting underserved smokers.

### Objectives

This paper describes the protocol for a randomized controlled trial (RCT) that will evaluate the efficacy and economic impact of a theoretically-based, fully-automated smartphone intervention targeting underserved smokers recruited through a large, local hunger-relief organization in West Central Florida. Participants (*N* = 500) will be randomized to 1 of 2 treatment conditions: 1) Standard Treatment (ST; *n* = 250) or 2) Automated Treatment (AT; *n* = 250). ST participants will be electronically connected to the Florida Quitline (Tobacco Free Florida). This approach is designed to mirror AAC, which was developed by our team to link smokers in healthcare settings with quitline-delivered treatment. ST will be compared to AT, a fully-automated treatment enrollment and delivery approach. AT participants will receive an interactive smartphone-based intervention including individually-tailored audiovisual and text content. Combination nicotine replacement therapy (NRT) in the form of transdermal patches and nicotine lozenges will be provided to all participants.

The primary aim is to evaluate the efficacy of AT in facilitating smoking abstinence. We hypothesize that at the 12-month follow-up assessment, self-reported smoking abstinence rates will be higher in the AT group (vs. ST). Secondary aims are to: 1) compare the magnitude of the mediated effects via common mechanisms (i.e., motivation, agency, stress/negative affect) on smoking abstinence between the ST and AT treatment groups and 2) conduct economic evaluations to evaluate the cost and/or cost-effectiveness of ST vs. AT.

Through these aims, we will determine if AT performs better—in terms of facilitating long-term smoking abstinence—than the ST approach. If efficacy is established, the AT approach will be scalable and easily implemented by community-based organizations.

## Methods

### Design overview

This study will utilize a 2-group RCT to compare the efficacy of ST vs. AT among 500 participants (250 per group). We will enroll up to an additional 20 participants (up to 10 per group) as part of a 12-week pilot study that will be conducted prior to the full trial.

**Fig. 1:**
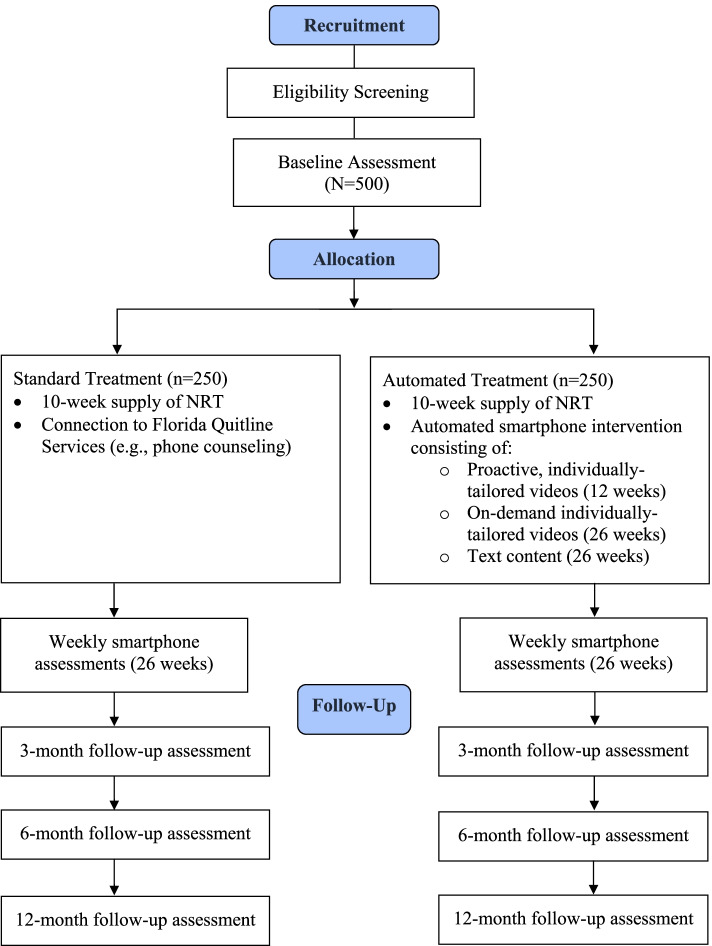
CONSORT diagram depicting trial design

Participants will be current smokers recruited from a network of food distribution centers in West Central Florida. Participants will complete screening, baseline, and 3-, 6-, and 12-month follow-up assessments in person, over the phone, or online using the Research Electronic Data Capture (REDCap) platform [30, 31]. Brief 4-item weekly smartphone assessments will be administered throughout treatment. Please refer to Fig. 1.

### Eligibility criteria

Inclusion criteria are: 1) at least 18 years of age; 2) English or Spanish speaking; 3) smoked at least 100 cigarettes in lifetime; 4) currently smoke at least 5 cigarettes a day; 5) willing to make a quit attempt within 1 week of enrollment; 6) possess a smartphone with a data plan and operating system compatible with the project app; and 7) have a valid email address. Exclusion criteria include the following: 1) medical condition that precludes the use of NRT; 2) current use of smoking cessation medications; 3) enrolled in another smoking cessation study; 4) household member enrolled in the study; 5) inadequate health literacy; and 6) failure to electronically confirm participation within 14 days of randomization via an electronic link.

### Recruitment and screening

We have developed a partnership with a large, local hunger-relief organization that is part of the national Feeding America network. Annually, this organization provides more than 95 million meals to nearly 1 million food insecure families in the 10-county area of West Central Florida. Study staff will recruit participants from affiliated food distribution centers using face-to-face, online, and remote (e.g., flyers, staff referrals) recruitment methods. Cigarette smoking prevalence in the general population is 13.7% [32], yet smoking rates are higher among individuals with low SES and the food insecure [2, 33]. Given the broad reach of this hunger-relief organization, we believe that our recruitment goal of 500 participants is attainable.

Study staff screen potential participants for eligibility in person, over the phone, or online. Eligible individuals who are interested in participating will provide informed consent in person, over the phone, or online, and they will receive a copy of the informed consent document. They will then complete a baseline assessment in REDCap. See Table 1 for a full list of measures.

**Table 1:**
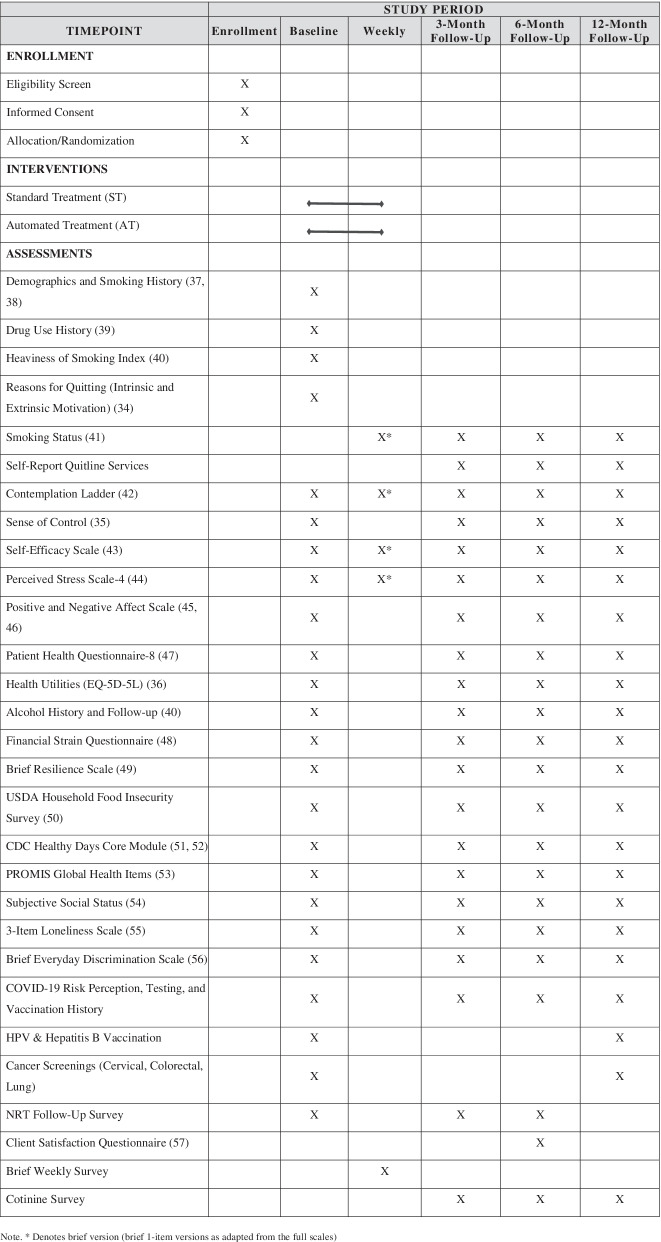
SPIRIT Flow Diagram of enrollment, interventions, and assessments

* Denotes brief version (brief 1-item versions as adapted from the full scales) [34–57]

### Randomization

Following completion of the baseline assessment, participants will be randomized to treatment group (ST or AT) using stratified randomization. Sex assigned at birth and nicotine dependence, as measured with the Heaviness of Smoking Index (HSI), will serve as the stratification variables.

### Participant tracking, compensation, and retention procedures

We will use several approaches to maximize follow-up. Baseline and the 3-, 6-, and 12-month assessments will be conducted in person, over the phone, or online at participants’ convenience via REDCap. In-person assessments will be scheduled to coincide with participants’ plans to visit food distribution locations. Participants will be compensated up to $160 (US dollars) for completing baseline and follow-up assessments (4 assessments x $40 = $160). We will also compensate participants for using their personal smartphones for study-related use (i.e., data, texting, minutes). Participants will be compensated for up to 26 weeks, based on the number of weekly assessments completed (26 weekly assessments x $10 = $260). They will also be compensated for returning cotinine tests at 3-, 6-, and 12-months (3 tests x $30 = $90).

We will also use the following procedures to reduce attrition: 1) reminder phone calls/messages delivered via the app, SMS, or by study staff prior to scheduled assessments; 2) providing multiple ways for participants to complete assessments (i.e., in person, over the phone, online); 3) scheduling follow-up assessments at participants’ convenience; 4) obtaining names, addresses, and phone numbers of 3 collaterals (i.e., relatives, friends); and 5) utilizing Whitepages.com to search for updated contact information.

### Conceptual framework

**Fig. 2:**
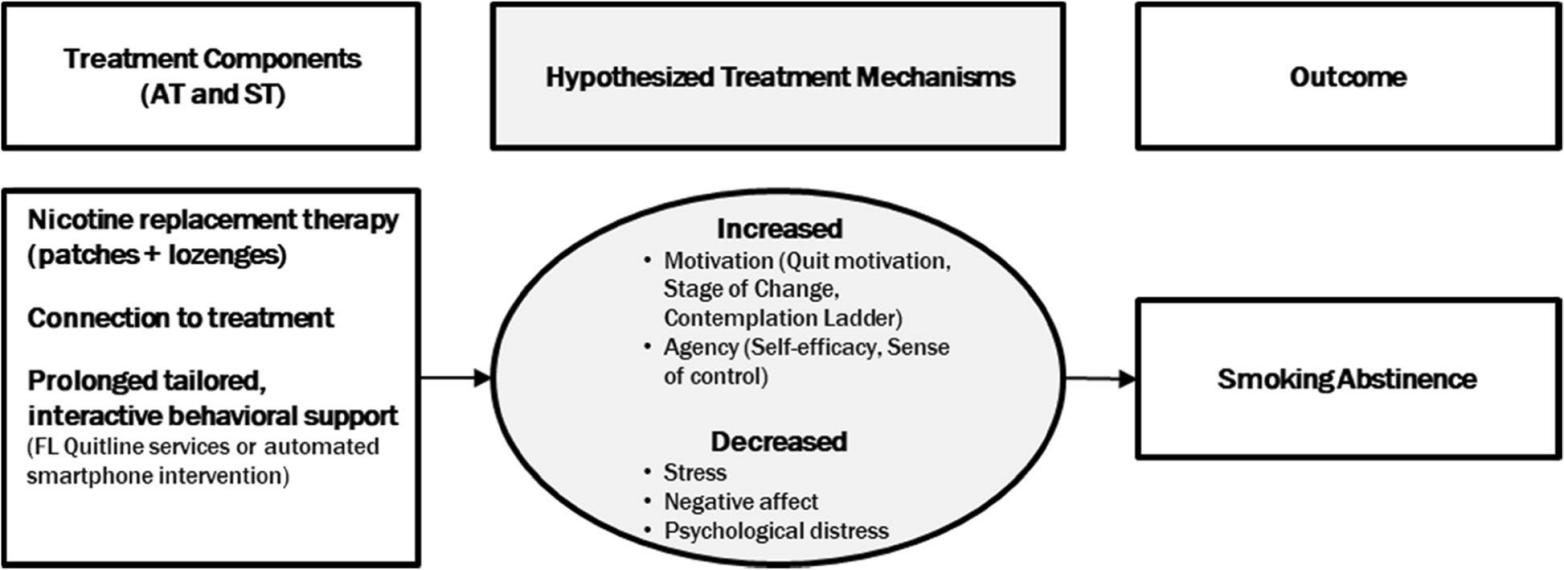
Hypothesized mechanisms underlying Standard Treatment (ST) and Automated-Treatment (AT)

Prior research and theory indicate that motivation and agency are mechanisms underlying smoking cessation treatment enrollment, and motivation, agency, and stress/negative affect are key mechanisms of quitting smoking [58–66]. ST and AT were designed to address these mechanisms. We hypothesize similar mechanisms between the 2 groups (ST vs. AT); however, we do not expect equal magnitudes of effect. A secondary aim is to evaluate the magnitude of the mediated effects between ST and AT. For example, ST participants might report more motivation to quit smoking, but AT participants may report higher self-efficacy. See Fig. 2.

#### Motivation

Motivation refers to one’s willingness or desire to change a behavior [67], and it plays a critical role in decisions related to behavior change and in the likelihood of achieving and maintaining change (see [58, 59]). Motivation is characterized by frequent fluctuations. In the context of smoking, over one-third (41%) of US smokers report daily changes in their motivation to quit smoking [68], and most quit attempts are unplanned [69, 70]. Shortly after quitting, motivation may weaken, and ambivalence may increase as individuals experience withdrawal symptoms and are exposed to high-risk situations and stressors [61]. Given its influences on the initiation of a quit attempt, cessation success, and the maintenance of abstinence, motivation should be targeted in treatment [38].

#### Agency (sense of control, self-efficacy)

Human agency**—**the ability to purposefully affect one’s behavior or life situation—is influenced by personal resources and situational factors [71, 72]. Concepts encompassed under agency include sense of control and self-efficacy [72]. Sense of control is the learned expectation that outcomes are not caused by chance, other people, or forces outside of one’s control, but rather, they depend on one’s personal choices and previous actions [41, 73]. Self-efficacy refers to an individual’s belief in their capacity to change a behavior [74], and it is dependent on context and behavior [73, 75]. Self-efficacy is among the strongest and most-studied predictors of smoking cessation treatment outcomes [63, 64, 76, 77].

#### Stress/negative affect

Stress-coping [78] and self-medication [79] models of substance use purport that people use substances to regulate mood and manage stress. Smokers often report smoking more when experiencing a negative mood (e.g., anxiety, sadness, anger, stress) and expect that smoking will help with managing these symptoms [66, 80, 81]. Additionally, there appears to be a dose-response relationship between stress/negative affect and smoking, such that greater distress is associated with higher nicotine dependence and heavier (vs. intermittent) smoking [82]. Regarding quitting, the magnitude and trajectory of stress/negative affect over time predict cessation [83, 84], as do individual differences in affective vulnerability [85, 86]. Research suggests that stress, negative affect, and psychological distress are associated with poorer smoking cessation treatment outcomes [77, 87–90], and most smokers report experiencing elevated negative affect after quitting, regardless of whether they use NRT [91].

#### Reciprocal relations among mechanisms

Reciprocal relations have been established among our key hypothesized mechanisms [61, 77, 92–94]. We hypothesize that ST and AT will reduce stress/negative affect via the following mechanisms: 1) reducing ambivalence and increasing motivation; 2) using coping skills training and problem solving to increase self-efficacy; and 3) using a holistic treatment approach to address other life concerns and stressors. We also expect that reductions in stress/negative affect will increase motivation and agency.

### Treatment groups

We created ST and AT to expand the reach of our existing evidence-based AAC model [7, 8] into the community. Both approaches involve the systematic assessment of smoking status (Ask) among individuals seeking food bank assistance, delivery of brief advice to quit to smokers (Advise), and an immediate offer of enrollment in treatment (Connect). Brief advice to quit will be delivered to all participants using a pre-recorded video clip. This automated approach to delivering brief advice to quit will standardize delivery across conditions, thus enhancing the future scalability and dissemination potential of AT.

We designed ST to mirror a clinic-based AAC model, as it consists of the standard Quitline-delivered services offered to uninsured smokers in Florida. AT will function as a fully-automated program capable of delivering smartphone-based treatment content that includes interactive messaging, images, and audiovisual clips. The AT intervention delivery period will span 26 weeks, with 12 weeks of proactive content and 26 weeks of on-demand access. All participants will receive a 10-week supply of combination NRT (nicotine patches and lozenges). Nicotine patches provide a low, constant dose of nicotine, which reduces nicotine withdrawal symptoms (e.g., physical symptoms, negative affect) after quitting, while lozenges provide a fast-acting dose of nicotine that helps to manage cravings. When combined with behavioral treatment, NRT effectively doubles the odds of quitting [95].

#### Standard treatment (ST)

After the baseline assessment and brief advice to quit video, we will provide participants with 10 weeks of nicotine patches and lozenges. We will connect ST participants with Florida Quitline services, where they will receive smoking cessation treatment delivered by the Quitline. Participants will complete weekly 4-item smartphone assessments for 26 weeks. Weekly assessments will consist of questions on smoking status, motivation, self-efficacy (to measure agency), and perceived stress (to measure stress/negative affect).

#### Automated treatment (AT)

Following the baseline assessment and brief advice to quit video, AT participants will be given a 10-week supply of nicotine patches and lozenges. AT will consist of: 1) 12 proactive treatment videos (delivered weekly) tailored on smoking status, motivation, agency, and/or negative affect/stress; 2) 26 weeks of on-demand access to treatment content; and 3) 26 weeks of text content.

AT participants will complete weekly 4-item assessments of smoking status, self-efficacy, motivation, and stress for 26 weeks. An algorithm will use participants’ responses to deliver brief (2–6 min) videos from a library of tailored content. See Table 2 for a list of weekly topics. The app will deliver the most suitable video each week, expressly tailored for each participant. During the first 12 weeks, weekly videos will be automatically launched after the completion of assessments. Referrals to other relevant treatment resources will be available through the app. These will include phone numbers for substance abuse treatment centers, psychiatric services, and social services.

**Table 2:** Automated Treatment session topic by study week

Week	Topic
Week 1	Preparing to Quit
Week 2	Quit Day
Week 3	What to Do if You Have a Cigarette
Week 4	Nicotine Patches and Lozenges
Week 5	Smoking, Stress, and Mood
Week 6	Health Benefits of Quitting
Week 7	Smoking and Your Weight
Week 8	Staying on Track
Week 9	Financial Benefits of Being a Nonsmoker
Week 10	Taking Care of Yourself
Week 11	Social Benefits of Being a Nonsmoker
Week 12	Life without Cigarettes

The AT app was designed to function autonomously and require minimal human involvement, while being suitable for implementation in various environments. The AT approach is expected to outperform but share common mechanisms of action with the current standard of care (i.e., quitline counseling). The app consists of both a staff-facing dashboard and a mobile application. Videos were created with the Adobe After Effects animation software package.

Based on our prior work and the extant literature supporting the efficacy of text message-based smoking cessation interventions, AT participants will also receive smartphone-delivered text content [24, 84]. This content will be delivered using notifications generated by the app. Participants will receive one message per day for the first 12 weeks and one per week in weeks 13–26. Notifications will start just before the scheduled quit date and will continue throughout the 26-week treatment period. Content was designed to target the hypothesized mechanisms, promote NRT adherence, and encourage participants to access on-demand content within the app. The text content will refer to participants by their first name to personalize treatment.

### Measures and assessment strategy

Several factors guided our assessment strategy. First, we attempted to include psychometrically-sound measures with established reliability and validity, and measures without established reliability and validity were required to have face validity. Second, measures had to either: 1) describe the sample; 2) predict smoking behavior; or 3) represent our hypothesized mechanisms. Notably, all the measures are based on theory, models of health behavior or nicotine dependence, and/or existing data. Third, to ensure a representative sample of smokers, we will recruit both English- and Spanish-speaking participants. The Florida Quitline is equipped to provide treatment in English and Spanish, and the mobile app (AT) will be available in both languages. Most of the measures were previously translated and validated in Spanish, and a certified translator will be used for measures that have not yet been translated. Finally, to reduce participant burden, participants will have the option of completing assessments in person, over the phone, or online. REDCap will be used to administer all assessments. REDCap is a secure, web-based app designed to support data collection. It will support the secure capture of data for cleaning, storage, and analysis. Moffitt Cancer Center is a member of the REDCap Consortium.

#### Primary outcomes

Our primary outcome is self-reported 7-day smoking abstinence at the 12-month follow-up. We will examine other definitions of abstinence (e.g., biochemically-verified abstinence using salivary cotinine, self-reported 24-h, 30-day, and continuous abstinence) as secondary outcomes. Participants reporting smoking abstinence will be mailed a saliva cotinine kit with instructions for providing the cotinine sample. Research staff will be available by phone and email to answer questions about the cotinine collection process. Participants will be asked to return cotinine samples using a prepaid envelope.

Regardless of treatment group assignment, participants will complete brief 4-item weekly smartphone-delivered assessments throughout the 26-week treatment period. AT participants will receive assessments via the app, and ST participants will receive assessments through a REDCap link via email or text (based on their preference). Assessments will be identical for ST and AT participants, but responses will be used to select appropriate treatment content for AT participants, as previously described.

#### Assessment of treatment engagement-related variables in AT

In addition to evaluating the efficacy of AT, we will track and examine the frequency and duration of AT participants’ app interactions. Interactions will be date and time stamped, and we will note the specific app features used by participants. This will allow us to examine the frequency and duration of participants’ utilization of various app components and the conditions under which they engage with components of the app. This information will guide future iterations of the app.

### Data analysis plan

Prior to inferential procedures, extensive descriptive analyses will be conducted to review variable distributions. In addition, descriptive statistics will characterize the sample and summarize the frequency and duration of participants’ interactions with the app. Exploratory analyses will examine how the utilization of various AT treatment components is associated with participants’ perceptions of the app and with cessation outcomes.

#### Primary aim

Our overarching aim is to evaluate the efficacy of AT on facilitating smoking abstinence. The primary outcome is self-reported 7-day point prevalence abstinence at the 12-month follow-up. We hypothesize that smoking abstinence rates will be higher in the AT (vs. ST) group. Using intention-to-treat methodology, we will code participants who do not complete follow-up as smoking. We will test this hypothesis using log-binomial regression, with intervention group as the primary predictor. We will control for relevant covariates (e.g., baseline nicotine dependence) and examine if treatment effects differ by sex assigned at birth.

Comparable analyses will evaluate the interventions’ effects on other definitions of smoking abstinence, including biochemically-verified abstinence using salivary cotinine and self-reported 24-h, 30-day, and continuous abstinence. We will also use smoking status at 3- and 6-months to evaluate the efficacy of AT. Generalized linear mixed models (GLMMs) with a log link function and the appropriate random-effect covariance structure will be used to analyze these data. Intervention, time, and their interaction will be included and tested in the model.

#### Secondary aim 1

We will evaluate the magnitude of the mediated effects via common mechanisms (i.e., motivation, agency, stress/negative affect) on smoking abstinence. Although we hypothesize common mechanisms of AT and ST, it will be important to pinpoint the relative strengths and weaknesses of the mechanisms in facilitating abstinence. These hypotheses can be tested using mediation analyses with intervention group (ST vs. AT) as the independent variable, abstinence at 12 months as the outcome variable, and the hypothesized mechanisms (motivation, agency, stress/negative affect) as potential mediators. To test these hypotheses, we will fit single and multiple mediator models, using the approaches of MacKinnon and colleagues [96] and Preacher and Hayes [97–99].

#### Secondary aim 2

We will conduct economic evaluations from a societal and a health system perspective to determine the cost and cost-effectiveness of both interventions. We will perform conventional cost-effectiveness analysis to summarize results as the incremental cost-effectiveness ratio (ICER; [100–102]). The ICER estimates additional resources needed to achieve an increase in units of effectiveness. The ICER is calculated as the difference in mean costs between a new (i.e., AT) and standard treatment (i.e., ST) divided by the difference in mean effectiveness between the treatments. The ICER is then compared with a published threshold value associated with a known cost-effective intervention. Two commonly-used measures—number of quitters and years of life saved (YOLS; [103])—will be used to compare the ICER with published cost-effectiveness studies. We will use data from the 12-month follow-up to calculate number of quitters for ST and AT. We will determine YOLS using a published algorithm that models YOLS per quitter [104]. This algorithm will incorporate recent estimates of age-specific smoking-related deaths [105]. We will also include quality-adjusted life years (QALY), which will be calculated using the EQ-5D-5L [47], a measure of health utilities.

We will use the net benefit approach to avoid the issue of zero denominators and to address uncertainty [106, 107]. This approach transforms the ICER into the net benefit, defined as NB(λ) = λ x ΔE – ΔC, where λ represents societal willingness to pay (WTP), ΔC represents incremental costs, and ΔE represents incremental effectiveness. We will report results both in terms of the conventional ICER and the cost-effectiveness acceptability curve—the latter can be used to inform decision makers of the probability that AT is more cost-effective than ST at various levels of societal WTP. This approach can be incorporated into a regression framework, which allows for covariate adjustments and the testing of interaction effects [108].

Finally, we will assess the short- and long-term economic impact of both interventions. For the short-term analysis, we will use number of quitters as the effectiveness measure and we will assess cost-effectiveness with data from the 3-, 6-, and 12-month follow-ups. For the long-term analysis, we will extrapolate the intervention effect to lifetime, using YOLS and QALY. Starting in the second year, a 3% discount rate will be applied to costs and outcomes. First, we will conduct deterministic analysis, where we will calculate point estimates of ICERs or cost differences. We will apply nonparametric bootstrapping methods to the person-level data to obtain 95% confidence intervals [109]. Next, we will conduct 1-way sensitivity analyses to elucidate the impact of alternative outcomes and measures of cost. We will then use a Bayesian approach to construct the cost-effectiveness acceptability curve and perform probabilistic sensitivity analysis [110, 111]. Bayesian analysis will be conducted with WinBUGS or STATA. Costs will be modeled as a gamma or lognormal distribution and abstinence as a binomial distribution. Finally, we will conduct regression-based cost-effectiveness analysis. Individual-level net benefit will be regressed on covariates, plus a binary variable reflecting intervention group (ST vs. AT). The model will be analyzed in 2 ways: 1) GLMM to examine cost-effectiveness over time and 2) Bayesian regression to systematically update information gathered at each time point.

#### Missing data and dropouts

Participants who do not complete follow-up will be coded as smokers. It should be noted that there are problems with this approach, particularly when comparing interventions with differential dropout rates [112]. Therefore, we will manage missing smoking status data using multiple imputation under the missing-at-random (MAR) assumption. We will also conduct pattern-mixture and selection models under the missing-not-at-random (MNAR) assumption [113].

#### Power considerations

Participants who do not complete follow-up will be classified as smokers for the primary analysis. We estimated power for self-reported abstinence between treatment groups at the 12-month assessment [114]. Based on findings from our AAC implementation study [9, 10], we expect an abstinence rate of 5% in ST. With 250 participants per group, a 2-group large-sample normal approximation test of proportions with a 2-sided 0.05 significance level will have 80% power to detect an increase in abstinence of 7% in AT vs. ST.

#### Data and safety monitoring plan

The MPIs will be responsible for all data monitoring and for compliance with all federal and institutional IRB policies and procedures for monitoring progress, safety, reporting of unanticipated problems or adverse events, and assuring actions resulting in suspension of the study are reported. All modifications to the protocol will be submitted for IRB approval. Summaries of all relevant discussions will be promptly disseminated to study personnel via e-mail, and retraining procedures will be implemented as needed. Appropriate modifications will be made in consultation with the designated program person at the National Institutes of Health if necessary.

All data collected will be kept confidential. Confidentiality will be protected by identifying all participants by ID numbers only, with all data stored and managed at Moffitt Cancer Center in a secure, HIPAA-compliant electronic database (REDCap). In addition, data storage, or data transfer if there is a request, will follow all Moffitt Cancer Center requirements for data security. When data sharing is requested, de-identified data files will be transferred on a password-protected and encrypted drive and will be maintained on institutional servers with appropriate antivirus software. Final de-identified data files will be maintained by the MPIs at Moffitt.

#### Dissemination plan

Study findings will be disseminated to the scientific community through presentations at local, national and international meetings and through peer-reviewed publications.

## Discussion

Smoking is the single largest behavioral contributor to disease, and it accounts for a significant proportion of socioeconomic disparities in the incidence and mortality of disease [2, 115]. Efficiently connecting underserved smokers to efficacious smoking cessation treatment is crucial for disease prevention and eliminating health disparities. Smartphone-based interventions have the potential to enhance the reach and efficacy of smoking cessation treatments, but the efficacy data are lacking—particularly among underserved smokers. This study is designed to address these gaps in the literature. It will consist of a 2-group RCT designed to evaluate the efficacy and economic impact of a theoretically-based, fully-automated smartphone intervention targeting underserved smokers recruited from a large hunger-relief organization serving West Central Florida. Participants will be randomized to receive either standard or automated treatment. ST will consist of treatment provided by the Florida Quitline, and AT will be comprised of an interactive smartphone-based intervention with individually-tailored audiovisual and text content. All participants will receive a 10-week supply of NRT. Our overarching aim is to evaluate whether AT performs better than ST in terms of long-term abstinence. We will also investigate potential mediators and conduct economic evaluations to evaluate the cost and/or cost-effectiveness of ST vs. AT. If successful, the AT intervention could be readily adopted and scaled up or down by various community-based organizations and social service networks.

A major design consideration was that the intervention should have the potential to make an important public health impact. Individuals with low-SES and racial/ethnic minorities are more likely to smoke, have limited resources for quitting, and be less likely to receive health care services [2, 3, 11, 12]. Thus, it is critical that these smokers are targeted directly within their communities. The AT intervention was designed to use modest resources and circumvent common treatment barriers. Moreover, we established a community partnership with a local organization supporting a network of food pantries serving individuals in the West Central Florida region. Participants will be recruited from food distribution centers using face-to-face, online, and remote recruitment methods. We hope to enhance participation by allowing screening, baseline, and 3-, 6-, and 12-month follow-up assessments to be completed in person, over the phone, or online, and brief 4-item weekly assessments to be completed via smartphones. Both interventions will be delivered remotely. ST will be delivered by the Florida Quitline, and AT will consist of a fully-automated smartphone-delivered intervention. We will leverage smartphone technology to enhance accessibility, improve treatment engagement, and reduce participant burden.

Limitations should be mentioned. First, all participants will be recruited from a network of food distribution centers in West Central Florida, and this may affect the generalizability of results. However, it should be noted that West Central Florida is quite large, as it is comprised of 10 counties, and the hunger-relief organization that we are partnering with provides more than 95 million meals to approximately 1 million food insecure families each year. Second, we require that eligible participants have a smartphone and an operating system compatible with the project app. Those who do not meet this criterion will be ineligible to participate. Given that smartphone ownership is widespread, and 85% of US adults own a smartphone [18], we do not foresee that this will be an issue. Moreover, we will compensate participants up to a total of $260 for up to 26 weeks to cover the costs of study-related smartphone use.

Theoretically-based, cost-effective, and sustainable cessation treatments with broad dissemination potential are needed. The AT intervention was designed to address this need. The scientific premise is built on the following: 1) smoking in underserved populations is a public health problem; 2) existing treatments may not address the needs (e.g., accessibility, efficacy) of underserved smokers; 3) partnering with community networks can reduce barriers and expand the reach of cessation services to underserved smokers beyond the healthcare system; 4) US smartphone ownership is widespread; 5) smartphone treatments have excellent reach and may be an ideal modality for underserved smokers; and 6) smartphone interventions have high potential for dissemination and sustainability due to their cost-effectiveness and extensive use.

## Data Availability

The datasets generated and/or analyzed during the current study will be available from the corresponding author on reasonable request.

## Abbreviations

AAC: Ask Advise Connect
AT: Automated treatment
Apps: Applications
EQ-5D-5L: Health utilities measure
GLMMs: Generalized linear mixed models
HSI: Heaviness of Smoking Index
ICER: Incremental cost-effectiveness ratio
MAR: Missing-at-random
MNAR: Missing-not-at-random
NRT: Nicotine replacement therapy
QALY: Quality-adjusted life years
RCT: Randomized controlled trial
REDCap: Research Electronic Data Capture
SES: Socioeconomic status
SMS: Short message service
ST: Standard treatment
US: United States
WTP: Willingness-to-pay
YOLS: Years of life saved
